# Evaluating the impact of ‘Ask the Specialist Plus’: a training program for improving cultural safety and communication in hospital-based healthcare

**DOI:** 10.1186/s12913-024-10565-4

**Published:** 2024-01-22

**Authors:** Vicki Kerrigan, Stuart Yiwarr McGrath, Cassandra Doig, Rarrtjiwuy Melanie Herdman, Shannon Daly, Pirrawayingi Puruntatameri, Bilawara Lee, Marita Hefler, Anna P. Ralph

**Affiliations:** 1 Menzies School of Health Research, Charles Darwin University, PO Box 41096, Casuarina, NT 0811 Australia; 2 Royal Darwin Hospital, Darwin, NT 0811 Australia; 3 Charles Darwin University, PO Box 41096, Casuarina, NT 0811 Australia

**Keywords:** Cultural safety, Training, Racism, Healthcare, First Nations, Australia

## Abstract

**Background:**

First Nations peoples in colonised countries often feel culturally unsafe in hospitals, leading to high self-discharge rates, psychological distress and premature death. To address racism in healthcare, institutions have promised to deliver cultural safety training but there is limited evidence on how to teach cultural safety. To that end, we created Ask the Specialist Plus: a training program that focuses on improving healthcare providers intercultural communication skills to improve cultural safety. Our aim is to describe training implementation and to evaluate the training according to participants.

**Methods:**

Inspired by cultural safety, Critical Race Theory and Freirean pedagogy, Ask the Specialist Plus was piloted at Royal Darwin Hospital in Australia’s Northern Territory in 2021. The format combined listening to an episode of a podcast called Ask the Specialist with weekly, one-hour face-to-face discussions with First Nations Specialists outside the clinical environment over 7 to 8 weeks. Weekly surveys evaluated teaching domains using five-point Likert scales and via free text comments. Quantitative data were collated in Excel and comments were collated in NVivo12. Results were presented following Kirkpatrick’s evaluation model.

**Results:**

Fifteen sessions of Ask the Specialist Plus training were delivered. 90% of participants found the training valuable. Attendees enjoyed the unique format including use of the podcast as a catalyst for discussions. Delivery over two months allowed for flexibility to accommodate clinical demands and shift work. Students through to senior staff learnt new skills, discussed institutionally racist systems and committed to behaviour change. Considering racism is commonly denied in healthcare, the receptiveness of staff to discussing racism was noteworthy. The pilot also contributed to evidence that cultural safety should be co-taught by educators who represent racial and gender differences.

**Conclusion:**

The Ask the Specialist Plus training program provides an effective model for cultural safety training with high potential to achieve behaviour change among diverse healthcare providers. The training provided practical information on how to improve communication and fostered critical consciousness among healthcare providers. The program demonstrated that training delivered weekly over two months to clinical departments can lead to positive changes through cycles of learning, action, and reflection.

 Over the last 5 decades or so, cultural awareness or cultural competency training has been offered to healthcare providers working in colonised countries. The training aspires to provide healthcare providers with important foundational information about First Nations cultures with the aim of improving how care is delivered and consequently reducing health disparities [1–3]. However, culture-based training has been extensively criticised for its ethnocentric agenda which can perpetuate ‘othering’ and negative stereotypes [4–7]. An alternative approach to upskilling health professionals to deliver care that is free from racism is required.

Cultural safety has been offered as a solution; proponents argue it can save lives [3, 8, 9]. While the term can easily be confused with the aforementioned training, cultural safety requires a critical examination of the mainstream institutions which were built to favour colonisers and places the onus for change on healthcare providers [10–12]. The urgent need to address these systemic issues came into sharp focus during the COVID-19 pandemic when First Nations peoples globally experienced high rates of infection and death [13, 14]. Cultural safety, developed in direct response to racism in healthcare can only be judged by patients, and is “about the analysis of power and not the customs and habits of anybody” ([8] p.181). Cultural safety should not be considered a moral imperative but instead recognised as a human right as envisaged by the World Health Organization constitution:

“The enjoyment of the highest attainable standard of health is one of the fundamental rights of every human being without distinction of race, religion, political belief, economic or social condition” [15].

Cultural safety requires providers and institutions to recognise and reflect on their own culture(s), and to ameliorate any actions which diminish, demean or disempower the identity of patients [3, 8]. Culturally safe health care means a patient feels emotionally, spiritually and culturally strong in their identity. Historically, this has not been a focus for hospital-based health providers. Traditionally providers have attempted to address poorer health outcomes among marginalised people by focusing on clinical care to address individual behaviour [13, 16]. However this approach tends to problematise the patient and does not recognise that the social determinants of health account for up to 70% of health outcomes and clinical care around 20% [16–18]. This is of particular significance in Australia’s Northern Territory (NT) where, due to colonisation and the related social determinants of health, First Nations peoples experience epidemic levels of chronic disease resulting in disproportionate rates of hospitalisation [7, 9, 19–21]. 

In Australia, First Nations peoples report feeling culturally unsafe in hospitals [9, 22–26]. Patients’ experiences of racism have contributed to high self-discharge rates, high levels of psychological distress, low rates of kidney transplantation, amputations without consent, and death [11, 25, 27–30]. Culturally unsafe care also contributes to healthcare providers experiencing burnout and professional dissatisfaction resulting in high staff turnover [31, 32]. In response, governments and medical colleges have committed to addressing racism in healthcare by ensuring care is culturally safe [33–35]. However, there is a lack of clarity on how to develop a workforce that can deliver culturally safe care hence policies have not translated into practice.

The practical application of cultural safety is twofold. One, healthcare providers must engage in critical self-reflection to develop their critical consciousness, which is a concept articulated by Freire in the seminal work “Pedagogy of the Oppressed” [3, 36]. Critical consciousness can be fostered when seemingly opposing parties engage in authentic conversations that challenge colonising ways of thinking [36]. Freire believed that dialoguing is an “act of creation” ([36] p.89) which leads to a view of “total reality” that considers all perspectives not just those of the oppressor ([36] p.108). Through dialogue those who identify with the dominating culture can develop an awareness of how that culture has created the historical, social, and economic factors that perpetuate inequity [3, 36, 37]. Studies in Australia have demonstrated that healthcare providers engage in critical thinking about Whiteness and privilege when they are presented with narratives shared by First Nations peoples that challenge stereotypical thinking and encourage an examination of power [38–40]. A critically conscious healthcare provider is better able to consider the social determinants of health, which have been identified as “the root causes of health inequities” [16]. Additionally, a critically conscious healthcare provider is willing to challenge their own position of power and privileged to change their practice and the systems they work in to deliver equitable healthcare [41]. Second, cultural safety focuses on developing a communication style that is effective, respectful and free from bias which manifests as racism. As doctors progress through medical training, communication skills decline as they are taught to suppress empathy and exert power over the patient, to control the patient interaction [42]. Additionally, language used by nurses and others has been found to dehumanise patients [43]. Adopting a culturally safe approach to communication means that healthcare providers reflect on their own learned communication practices and adjust to avoid demeaning patients [8, 44, 45]. By changing their communication style, healthcare providers can create an environment where power is shared between patient and provider, resulting in a culturally safe clinical consultation [44]. 

Understanding cultural safety praxis as described above we developed a communication and cultural safety training program called Ask the Specialist Plus. It is an 8-week program based on the podcast ‘Ask the Specialist: Larrakia, Tiwi and Yolngu stories to inspire better healthcare’ [46]. The podcast consists of 7 episodes in which Larrakia, Tiwi and Yolngu leaders, referred to as the Specialists, answer hospital-based doctors’ questions about working with First Nations patients. Whilst the podcast was designed to be deliberately local, universal truths applicable beyond the NT and outside of healthcare were apparent [47]. An evaluation of the podcast found the Specialists ‘counterstories’ [37], which challenged stereotypes and asserted First Nations voices and perspectives on health, encouraged the development of critical consciousness among doctors who reported positive attitudinal and behavioural changes after listening to the podcast. The ‘Plus’ refers to facilitated discussion groups that build on podcast episodes. The design moves away from one-off training which is perceived to be an institutional tick-box exercise, to a model that encourages healthcare providers to critically reflect on their own thoughts and practices through ongoing group discussions [48]. 

Ask the Specialist Plus was piloted in the NT at RDH in 2021. The aim of this study is to assess the feasibility and acceptability of the Ask the Specialist Plus pilot training program by analysing survey responses from participants who completed the program. This evaluation seeks to evaluate both the delivery mode and the content of the training program.

## Methods

### Study design

The development and evaluation of Ask the Specialist Plus was nested in a larger Participatory Action Research (PAR) [49] project called the Communicate Study Partnership [50]. The Study aimed to improve First Nations peoples experience of hospitals in the Northern Territory by exploring barriers and opportunities to support culturally safe communication practices. Philosophically, the Communicate Study was shaped by the constructivist concepts of cultural safety, Critical Race Theory and Freirean pedagogy [8, 36, 37]. Cultural safety, CRT and Freirean philosophy focus on redressing the power imbalance between the hegemony and marginalised peoples by encouraging perspective taking amongst the hegemony through dialogue. When implemented in the healthcare context in Australia, these decolonising philosophies support healthcare providers to develop critical consciousness by engaging in dialogue with marginalised peoples [51]. These decolonising philosophies advocate for elevating marginalised voices as a solution to racial disparity and recognise that the descendants of the coloniser and colonised share responsibility to dismantle racism. These philosophies underpinned the design of the training, how the survey was constructed and the qualitative analysis.

### Researcher backgrounds

Aunty Bilawara Lee is a Larrakia Elder, a healer and teacher with more than 50 years’ experience in education, health and the community sector. Pirrawayingi Puruntatameri is a Tiwi Elder with 40 years’ experience working in health, education, justice and the community sector. Rarrtjiwuy Melanie Herdman is a Gälpu woman from the Yolŋu nation. Her work spans the health, environmental, political and research sectors. Stuart Yiwarr McGrath is a Gumatj man from the Yolŋu nation; he is an Aboriginal Health Practitioner, a student of nursing and researcher. Shannon Daly, from Darwin, was trained as an Aboriginal Health Practitioner before becoming the Consumer and Cultural Consultant at RDH where she delivered cultural advice and training to staff. Vicki Kerrigan is an Australian born English speaking White researcher of Anglo-Celtic heritage, communication researcher and former radio broadcaster. Cassandra Doig is an Australian born English speaking junior doctor of Polish and Scottish heritage. Academic supervisors Anna Ralph and Marita Hefler are White researchers who have extensive history working collaboratively with Aboriginal peoples and organisations on health issues. Anna Ralph is also an infectious disease medical consultant at RDH.

### Study setting

Conducted on Larrakia country, this pilot was embedded in a Participatory Action Research (PAR) [52, 53] project exploring opportunities to improve culturally safe communication at RDH [54]. RDH is a 360-bed hospital managed by NT Department of Health where approximately 70% of inpatients identify as First Nations and many speak English as a second language [33]. Highlighting the cultural and linguistic diversity, 15 Aboriginal languages were documented as being in use over 4 weeks in just one ward [22]. Less than 10% of NT health staff identify as First Nations; many RDH staff, from southern Australian states or overseas, come to Darwin for “a short time or a good time” [32]. This pilot training was offered in addition to standard RDH cultural awareness training which is offered to all new employees as either a one-day face-to-face workshop or online modules. At the time of the pilot, the proportion of staff who had completed RDH cultural awareness training in a one day workshop or online modules was 10% and 20% respectively [55]. 

### Training design

Ask the Specialist Plus was piloted in two departments: Obstetrics and Gynaecology (O and G) and Endocrinology (which primarily delivers diabetes treatment services). Departmental leaders requested the training after recognising the need. Hospitals are known to be resistant to change [56], hence leaders who can act as change agents are vital to the success of implementing unique innovations [57]. Additionally when seeking to support anti-racism praxis it is crucial and “also efficient to start with one’s own immediate spheres of influence” ([58] p.3).

Ask the Specialist Plus, offered during set weekly teaching times, consisted of 8 × 1-hour discussions delivered over consecutive weeks to departments. Training embedded in allocated teaching ensured staff had protected time away from clinical duties and showed staff that cultural safety and communication training was valued by leaders as much as other clinical teaching. Participation was open to all members of each department with a cap of 25 participants per session to encourage sharing of ideas. Group sizes for each session are listed in Table 2. The number of sessions per participant varied. Because surveys were anonymous, we were unable to document the consistency of attendance or collate responses according to individual. The podcast evaluation [47] revealed some doctors were confronted by some perspectives shared by Specialists, hence facilitators worked to create a supportive teaching environment while keeping in mind that teaching cultural safety should be “uncomfortable but comfortable enough” to inspire growth [59]. 

The first session was an introduction to cultural safety which clarified the difference between cultural awareness training and cultural safety training. In subsequent weeks staff were asked to listen to one podcast episode per week prior to each face-to-face session (Table 1), which was designed to be a catalyst for the discussion. Facilitators (VK, SD) prepared PowerPoint slides that included discussion prompts such as peer-reviewed publications, books and videos that supported perspectives shared by the Specialists. The Specialists were Larrakia, Tiwi and Yolŋu leaders: Aunty Bilawara Lee, Pirrawayingi Puruntatameri, Rarrtjiwuy Melanie Herdman, and Stuart Yiwarr McGrath. Staff from the NT Aboriginal Interpreter Service also shared expertise.

**Table 1:** ‘Ask the Specialist Plus’ training program weekly discussion topics

Week	Topic	Podcast Episode
1	Introduction to cultural safety	N/A
2	Get to know your patient	Episode 1
3	Communicating with your patient	Episode 2
4	Communicating with interpreters	Episode 3
5	Patient centred care	Episode 4
6	Informed consent	Episode 5
7	Recognising and addressing racism	Episode 6
8	Perspectives on health and wellbeing	Episode 7

Specialists changed each week: this was to ensure a diversity of perspectives and meant the burden of teaching was shared among Specialists who had extreme workloads. The training team included a White facilitator (VK) who attempted to model culturally safe communication. The diverse team model recognizes that racism is a relational issue that requires both the descendants of the colonisers and the colonised to collaborate in its dismantling [36]. It is not uncommon for White individuals to become defensive, disengage, and shift the responsibility for addressing inequities to those who experience racism. To overcome such challenges, a White facilitator can assist White students in managing their discomfort and a diverse team can also share the emotional and mental challenges involved in unpacking racism [60–62]. 

### Survey data

Anonymous paper-based surveys were completed at the conclusion of each training session. Using five-point Likert scales, participants ranked the quality of training across the following domains: clinical relevance; relevance to Royal Darwin Hospital work; bias and stereotypes challenged; behaviour change inspired by the training; training format; facilitator style; and training duration. Surveys contained space for free text comments. Participants were asked to identify their professional role however some did not and therefore are referred to as ‘staff’ or ‘attendee’ in the results. Age, gender and ethnicity data were not collected.

### Data analysis

Data from each training session were collated by VK, scanned and saved on a secure server. CD entered data into an Excel spreadsheet, grouped by department and week. For each domain, the proportion of responses for each score (1 to 5) was calculated. The proportions were then averaged across all weeks within each department. Free text comments were collated in a Word document and grouped according to week. VK uploaded documents to NVivo12 to deductively analyse data following Kirkpatrick’s training evaluation model [63]. The model evaluates training across four levels: 1) reaction to training; 2) learning; 3) on the job behaviour change and; 4) observable organisational results. Data relating to level 4 were not collected and therefore excluded from analysis. Preliminary analysis was reviewed by SYM, CD, BL and APR and results were further refined. A draft manuscript was presented to all coauthors who provided feedback which was incorporated into the final manuscript.

### Ethical considerations

Approval to conduct the study was provided by the NT Department of Health and Menzies School of Health Research Ethics Committee. Training attendance and survey completion were voluntary.

## Results

Fifteen Ask the Specialist Plus training sessions were delivered to the O and G and Endocrinology departments at RDH between March and October 2021. A total of 171 surveys from both groups were completed; 73% of participants across all sessions completed the survey. Participants included medical students, doctors (interns, registrars, consultants, locums), midwives, allied health professionals and nurses. Weekly participation numbers were relatively well sustained throughout delivery but with a slight downward trend over 8 weeks (Table 2).

**Table 2:** Survey completion rate and number of attendees

Training topic	Survey completion/No. attendees (%)	
	Obstetrics and Gynaecology dept. (O and G) 17/3/21 − 5/6/21	Endocrinology dept. 25/8/21–6/10/21
WK1: Introduction to cultural safety	16/20 (80%)	14/19 (74%)
WK2: Get to know your patient	8/11 (73%)	13/17 (76%)
WK3^a^: Communicating w/ patients	14/16 (88%)	11/19 (57%)
WK4^a^: Communicating w/ interpreters	6/8 (75%)	
WK5: Patient centred care	13/14 (93%)	14/19 (74%)
WK6: Informed consent	12/15 (80%)	11/19 (58%)
WK7: Recognising and addressing racism	10/12 (83%)	7/15 (46%)
WK8: Perspectives on health and wellbeing	15/15 (100%)	11/16 (69%)

^a^Due to impact of COVID-19 on hospital operations, training sessions “Communicating with patients” and “Communicating with patients and interpreters” were merged

### Ranking teaching domains

**Fig. 1:**
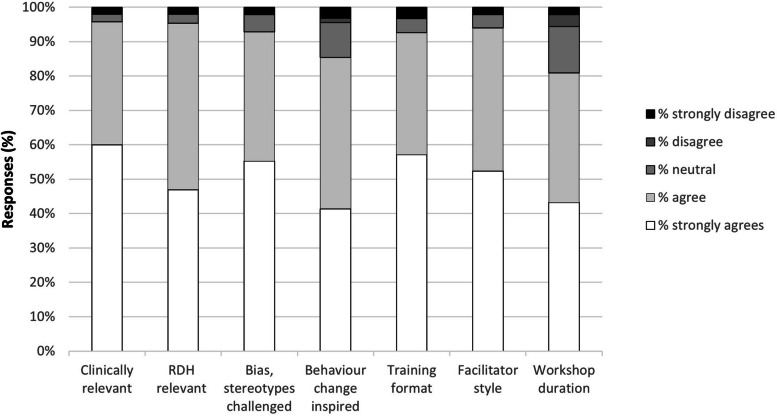
Survey responses summarised for all training weeks, Obstetrics and Gynaecology and Endocrinology departments at RDH, 2021

The mean overall assessment score for the Ask the Specialist Plus was 4.5 out of 5 (range of means scores: 4.2 [duration of sessions] to 4.7 [clinical relevance of sessions]). Scores across the 7 teaching domains were high (Fig. 1) revealing attendees agreed or strongly agreed the training was relevant and inspiring. Two surveys appeared to have been completed inaccurately since scores of 1 (worst score) on the Likert scale was selected for all responses yet free text responses were positive, but results were included with the scores as shown.

### Free text comments from surveys

Free text comments provided deeper insights into participant experiences. Surveys contained 275 free text comments which ranged from single word answers to a 41-word paragraph. Due to the study design, results pertain mainly to Kirkpatrick’s level 1 and 2 categories. We have adapted level 3 to include feedback relating to planned or, in later weeks of the training, reported behaviour change, not observed behaviour change. Kirkpatrick’s levels are used to provide the structure to methodically present results.

*Kirkpatrick’s Level 1* encompasses participant engagement and satisfaction, responsiveness to the format and facilitator, interest in the subject matter and motivation to acquire knowledge.

Attendees reacted positively to the training, with many stating it was an unfulfilled need. After attending the introduction to cultural safety, one Endocrinology attendee said they were grateful “something is being done” because “I have a lot to learn” and an allied health professional said: “we can always do better”. Many also commented that they appreciated learning the difference between cultural awareness and cultural safety. Words used to describe the training included: inspiring, relatable, vital, thought-provoking, useful, practical, transformative, and confronting.

Attendees enjoyed the format of listening to a podcast episode which primed attendees for a weekly 1-hour discussion. An endocrinologist described the podcast as “helpful and impactful” and an O and G medical student enjoyed the flexibility of the podcast which allowed them to listen and reflect “when I am walking/driving time”. However, three weeks into the 8-week course, one O and G registrar revealed “I haven’t listened to the podcast yet” which may indicate lack of interest, dislike of the format or lack of time. The 1-hour discussions over consecutive weeks were valued in comparison to online training. Free text comments supported the Likert responses: 19% of attendees ticked ‘neutral, disagree or strongly disagree’ when asked if the workshop duration was ‘perfect’. This was the highest neutral and negative response to any question. Attendees also enjoyed meeting weekly which allowed time for reflection, action and more reflection.

*“I have found that in between the sessions I have thought more about these issues. I am deepening my understanding because of the opportunity to discuss and most importantly listen and feeling more ready to change my practice.”* - O and G doctor, WK 7: Recognising and addressing racism

Facilitators were perceived as knowledgeable, supportive, and engaging. After joining “Recognising and addressing racism”, an endocrinologist said the White facilitator and Specialists made them “feel safe talking about a difficult topic”. Attendees appreciated advice and practical tips from the Specialists who shared real-life examples and sometimes differing perspectives. An O and G doctor who attended 5/8 sessions wrote:

*“Having face to face conversation with Elders and Mel and Stuart is INVALUABLE. You can’t get this transformative impact without meaningful discussion and engagement.” –* O and G doctor, WK 8: Perspectives on health and wellbeing

When asked to address a survey question regarding most impactful topic, the same O and G doctor wrote: “I can’t choose. They all changed the way I think, reflect and behave.” Most attendees said training should be delivered to all staff. In the final week of the endocrinology training, a consultant wrote:

*“I would strongly recommend this being integrated into all areas of health – junior and senior staff. Incorporating reflective aspects of coming together weekly has been very powerful.”* – Endocrinologist, WK 7: Perspectives on health and wellbeing

Some expressed concerns. An O and G medical student, after attending a session on patient centred care wrote the session was “Confronting. Makes me think.” Also after attending a session on informed consent in which content included examples of failures to obtain informed consent, an O and G registrar suggested healthcare providers should be recognised for good intentions and content should focus on solutions:

*“Majority of doctors/midwives (in our field at least) want patients to have the best communication, understanding but topics seem to focus only on the fault of the communication of the health professional, not practical solutions around the systems that prevent communication.”* – O and G registrar, WK 6: Informed consent

*Kirkpatrick’s Level 2* covers the development of new skills, the acquisition of knowledge, and the manifestation of a shift in attitude because of the training.

We found attitudes towards investing time with patients and families changed as attendees became aware that building trust by listening to patients talk about their social and cultural circumstances can improve health outcomes. An O and G obstetrician summarised how to create trust: “Introduce yourself. Learn language. Listen.” Attendees learnt techniques to establish rapport in the fast-paced hospital: be willing to share something personal, give the patient time to share their story beyond the medical presentation and ask the patient to teach them a word or two in their language. Relating to the importance of attitudes, many attendees wrote down the words “be genuine” after listening to podcast episode 2 in which Specialist Pirrawayingi Puruntatameri said that a genuine attitude towards respectful interactions can improve communication. Over the course, attendees developed a deeper understanding of what one endocrinology attendee referred to as “reasons for non-compliance”. They learnt that the absence of Aboriginal Liaison Officers, interpreters and/or family members contributed to a “lack of trust” which can lead to patients disengaging. They also learnt about historical segregation policies which resulted in “native wards” for First Nations patients and how history contributes to intergenerational trauma and a distrust of health services. Indicating development of critical consciousness, an O and G midwife said they will work to create a “less medicalised” environment to reduce patients’ reluctance to come to hospital because they now have a better understanding of “how patients feel coming into hospital”.

After attending the introductory session on cultural safety which explored the clash between hospital systems and culturally safe healthcare, an O and G doctor said the hospital operates like a “factory” which expects patients to “fit in” and committed to “allowing more time for talking”. Time pressure to move through patients quickly to satisfy hospital expectations was repeatedly mentioned as a barrier to culturally safe communication:

*“Need more time to allow patient empowerment and time to talk and adequate informed consent. This is very difficult, we really struggle to find time to allocate to patients and constantly take shortcuts with discussions.”* – O and G doctor, WK 6: Informed consent

The value of working with Aboriginal Liaison Officers and interpreters became apparent and many recognised both professional groups were undervalued and underused by healthcare providers. A consultant from O and G and another from Endocrinology assumed non-clinical First Nations professionals were “not needed” however after the training both committed to working with First Nations non-clinical staff. Another O and G consultant displayed a nuanced understanding of the need to collaborate with First Nations professionals whilst also recognising their workload:

*“Draw on resources as appropriate but don’t overburden people with knowledge. Take the opportunity to further my own capabilities working with cultural safety”.* – O and G consultant, WK 1: Introduction to cultural safety

Regarding communication, attendees learned that differences in body language and eye contact is related to cultural norms and should not be misconstrued as rudeness or disengagement. Attendees developed a new appreciation for communicating with patients in their first languages and for the challenges patients face if they do not speak English as a first language. An O and G consultant reflected on “the complex cognitive processes of speaking in 2nd language”. An endocrinology attendee said they realised “just how little patients understand”. Another endocrinology staff member admitted they “rush through the day” and communication is “left behind”; henceforth they committed to “booking interpreters ahead of time.” Attendees learned practical skills such as how to book an interpreter and also learned that it is the healthcare provider, not the patient who requires the interpreter because the health provider does not speak the patient’s language. A medical student with the Endocrinology department indicated increased confidence regarding how they could contribute to improving culturally safe communication: “From the start, try to arrange an interpreter for a patient rather than wait for a senior to suggest it”.

Attendees also learnt about the importance of respecting perspectives on disease causation and cure that may not align with biomedicine. For example, the Specialists spoke about the importance of spiritual healing which was new knowledge for many. In response to learning about the importance of a stillborn foetus being buried on country, an O and G registrar said: “I will explore the need for my patients to have their cultural needs met eg. returning pregnancy to them after termination”. Indicating a desire to learn more, an Endocrinologist wrote:

*“Really important session today, would be useful to expand into additional cultural awareness training around different spiritual and cultural beliefs around the Top End.” –* Endocrinologist, WK 7: Perspectives on health and wellbeing

Attendees were introduced to new concepts such as ‘weathering’ (the cumulative effect of microaggressions) which can influence an individual’s capacity to trust healthcare providers. Subsequently an Endocrinologist said they had a “greater awareness of power imbalance and greater awareness of the potential numerous negative experiences that may be impacting the interaction.” Attendees learnt that to cope with racism, patients may appear to give consent even when confusion remains. As one endocrinology attendee said: “saying yes does not always mean the patient gives consent.” To overcome issues regarding uniformed consent, ‘teach back’ was discussed as a way to ensure effective communication. Reflecting on using ‘teach back’, an O and G consultant said:

*“Aboriginal patients say “yes” to protect us from shame, not themselves. Aboriginal patients don’t complain or suggest feedback, making it more important to be proactive. Think ‘outside the box’ regarding problem and solution”.* – O and G consultant, WK 4: Communicating with interpreters

Attendees developed a better understanding of bias, White culture in healthcare, interpersonal racism and the power imbalance between patient and provider. After attending “Recognising and addressing racism” many committed to reflecting on stereotypical thinking and how it affects patient interactions. An O and G registrar said they became aware of their “own mistakes that I wasn’t aware I was making before” such as assuming individuals from marginalised cultures can speak on behalf of their entire racial group. An O and G doctor who had attended 4 out of 7 available sessions wrote:

*“Rather than feel guilty and impotent in addressing my white privilege and maleness, focus on addressing perceived power imbalances in my interactions”* – O and G doctor, WK 7: Recognising and addressing racism

Only one attendee expressed confidence discussing racism. After attending “Recognising and addressing racism” an Endocrinology team staff member who had attended all previous training sessions wrote: “Been many years since I realised that I was a racist whilst studying this subject” and requested the training include “more examples of growing up as a racist”. Another attendee from Endocrinology wrote “This should be a compulsory education session for all the healthcare providers working/joining Top End health”. In contrast an O and G attendee said, “The discussion was thought provoking however I’m not sure of what changes I can make on a day-to-day basis in my interactions”.

*Kirkpatrick’s Level 3* has been modified to include feedback relating to anticipated or reported behaviour change, not observed behaviour change as per Kirkpatrick’s original framework.

On training completion, consultants from both O and G and Endocrinology committed to incorporating culturally safe communication practices into the standards of care. But an Endocrinologist also articulated that the “frustrations of working within an institutionally racist system” may hamper progress.

Working with interpreters was a popular option for intended behaviour change. Attendees committed to not using family as interpreters and booking interpreters ahead of time when appropriate. An Endocrinologist said they will “work hard to improve systems to enable an interpreter to be present in my weekly telehealth clinic”. After attending the “informed consent” discussion, an O and G consultant planned to work with interpreters to record explanations of common procedures such as caesarean sections to ensure consent was informed.

Individually, attendees committed to making small changes which they believed could have a big impact on patient experience. Many committed to wearing clothes with Indigenous prints to show they were an “ally”, using a map of NT languages as a visual tool to assist with language identification and learning a few words in the patient’s first language to build rapport. After reflecting on the perception that stethoscopes can be considered a symbol of power, an Endocrinologist said: “I will think about the power imbalances when interacting with patients. Practical things such as taking off my stethoscope!”.

To improve communication, attendees committed to changing some of the standard phrases used. After the session on “Communicating with interpreters” many said they would replace the phrase “Do you speak English?” with the alternative “What language do you speak at home?”. They learnt the latter is designed to encourage the patient to share their first language which may lead to engaging an interpreter. During the “patient centred care” discussion, staff workshopped alternatives to the closed question often asked at the end of consultations, “Do you have any questions?”. As an alternative many committed to asking open ended questions to encourage discussion and clarify understanding. Although one O and G attendee expressed concern: “It is well ingrained in my practice to say at the end ‘Do you have any questions?’. I’m working hard not to”.

### Ideas to improve training

Most said the “great” training should be mandatory and many requested “ongoing refreshers”. Every week one or more of the attendees requested longer sessions to allow for more discussion to encourage group reflection. An O and G medical student requested that the training provide: “More stories on moments of success/failure in communication between HCP’s and patients from diverse cultures” (*HCP = healthcare provider*). Secondly, practical tips and resources to improve communication such as First Nations language maps and apps were requested. Thirdly, staff wanted to discuss real life case studies and one Endocrinology team member suggested, “Bring a case that has worried us and discuss how we could have changed an outcome”. Another attendee from Endocrinology suggested “role play with volunteer Indigenous actors”.

Indicating a desire to critically reflect, one attendee from the Endocrinology department suggested “Pre intro questions about our thinking – so we can review our self-reflections from time to time” and an O and G attendee wanted to be observed during patient interactions. Finally, O and G staff requested a podcast and discussion specifically about women’s health because as a registrar stated it is “Naturally an area where patients of all cultures are vulnerable and emotive”.

## Discussion

The Ask the Specialist Plus training aimed to support culturally safe communication by developing healthcare provider critical consciousness. Critical consciousness was fostered by opportunities to converse with First Nations Specialists and facilitated discussions which encouraged attendees to critically reflect on their own attitudes and behaviour in the context of colonised Australia. Quantitative results indicate 90% of participants agreed or strongly agreed the training was valuable across the seven domains assessed. Students through to senior staff learnt new skills, stereotypes were challenged, and individual bias and institutionally racist systems were discussed. Attendees recognised previous mistakes and expressed confidence regarding small but impactful changes they could make to their own behaviour to create healthy environments for safe and effective communication. Some senior staff also committed to addressing the institutional barriers that restrict individuals’ abilities to deliver culturally safe care. Some staff indicated they were unable to attend every training session but comments still indicated growth in thinking during the 2 months. Delivering training over several weeks in which key concepts are reinforced meant that staff who needed to prioritise clinical demands or accommodate shift work could still participate in some of the program. We have provided evidence that training delivered during working hours to departmental teams, which included diverse professions and levels of experience over consecutive weeks, can encourage cycles of learning, action and reflection which leads to positive change.

Ask the Specialist Plus was piloted at RDH because of a commitment from departmental leaders. By collaborating with middle management, our aim was to influence systems change because while systems are complex “they are people-made and people-run” and so can be transformed by people at all levels ([64] p.186). The health workforce can contribute to eliminating health inequities if they are supported by engaging training programs which take action towards racial equity [58]. While the training package was site specific because racism manifests differently according to geographical contexts [65, 66] the techniques used could be adopted in any jurisdiction. Issues regarding culturally unsafe care and poor communication are also experienced by minority populations and Indigenous peoples globally who are profiled by health systems built on White and/or dominant culture norms [19, 67–70]. 

This evaluation of a pilot training program provides preliminary evidence of beneficial impact of cultural safety training. This fills an important gap highlighted in previous literature regarding the lack of evidence of impact of cultural safety training [1, 30, 71]. By listening to the Specialist counterstories, which challenged the deficit narrative by offering alternative perspectives which have been silenced by colonisers [72, 73], staff identified and critically reflected on the Eurocentric beliefs and practices that dominate healthcare. Healthcare providers who have capacity to adopt such cultural humility can contribute to reducing First Nations peoples’ fear and avoidance of seeking timely healthcare services [9]. We have also contributed to evidence that racism is best cotaught by educators who represent racial and gender differences [74]. This ally-ship model overturns the assumption that tackling racism is the responsibility of those who experience it: “racism is a relationship in which both groups are involved” ([75] p.64). The co-facilitation model may also reduce the risk of harm and violence First Nations facilitators can experience when teaching cultural safety [62]. 

Two findings deserve specific attention. Firstly, healthcare professionals want more training to improve culturally safe communication. This NT specific request is echoed globally by First Nations patients and staff and healthcare providers in other colonised countries who also recognise this need [6, 11, 22, 26, 76–79]. For most staff, attending Ask the Specialist Plus was the first time they had committed to, and connected with, training which was previously perceived as a tick-box exercise with no relevance to delivering clinically safe healthcare. In an era when online training modules are considered satisfactory and economically prudent, we argue there is value in investing in pedagogical practices which contribute to positive change [80, 81]. Our research provided evidence that counterstories [37] from First Nations Elders, interpreters and leaders can disrupt the dominance of Whiteness in healthcare, thereby contributing to the creation of a culturally safe hospital service. The format increased opportunities for staff to have positive contact experiences with First Nations people outside of the stressful clinical environment. Increasing positive contact experiences, a key feature of anti-racism training, generated empathy and encouraged perspective taking which assisted healthcare providers to develop their critical consciousness: this is the personal development work required to address racism in healthcare [66, 82, 83]. We encourage cultural safety trainers to invest in training formats such as podcasts, videos, virtual reality experiences and face to face workshops. While managers consider didactic online training modules to be cost effective and time efficient, healthcare providers wishing to improve the delivery of culturally safe care perceive online training as an ineffective time-wasting exercise that contributes to loss of collegiality and merely ticks boxes for institutional reports [32, 47, 84, 85]. Good retention at Ask the Specialist Plus over the weeks of program delivery, despite extreme pressures of clinical work compounded during the periods of delivery by COVID measures, attest to the level of interest and appeal of the program.

Secondly, the receptiveness of staff to discussing racism was noteworthy considering racism is commonly denied in healthcare [29, 81, 86]. Health staff who call out racism are often silenced, ignored or alienated [9]. Displays of White fragility and colour blind racism by White managers are used to justify inaction in the face of inequity [75, 87]. Resistance to discussing racism also exists because individuals lack racial literacy [88, 89]. Through Ask the Specialist Plus, staff became aware of the everyday nature of racism, they learnt about White culture and how they can contribute to anti-racist practice in healthcare. Some displayed capacity to be ‘truth tellers’ willing to examine both interpersonal and institutional racism [90]. Improving racial literacy among healthcare providers may reduce bias when interacting with marginalised peoples [91]. Globally, as a result of anti- racism efforts and movements such as #BlackLivesMatter there has been a decline in overt manifestations of racism; however the “attitudes, behaviours and underlying institutional structures” that perpetuate racism remain [66]. To dismantle oppressive systems we must engage with and document experiences of racism amongst racial and ethnic groups [92]. We must also document projects such as this that offer solutions, small or large, to the seeming intractability of racism [93]. Racism mutates like a virus: it evolves according to political, geographic, economic and social circumstances to ensure its survival [88, 94, 95]. While the instability of racism means it is challenging to define it also means it can be modified.

Regarding research limitations, we recognise that quantitative surveys can overestimate participant satisfaction, hence space for free text comments was added for each quantifiable question. We also recognise that free text comments can be unreliable: some have found that comments in anonymous evaluation surveys tend to be more positive than negative whereas other research has found comments to be highly critical and counterproductive [96–98]. The surveys we received contained a higher number of positive comments. We have reported the full range of comments including negative feedback. Another limitation related to potentially incorrect scoring in which a mismatch with written comments was evident, which may have falsely reduced the reported scores. As the surveys were anonymous it was impossible to verify the participants’ intentions. We subsequently improved the survey to reduce the risk of inadvertent errors by including happy through to unhappy emoticons to illustrate the Likert scale. Additionally, observable behaviour change was not documented in this pilot project nor was any impact on organisational results as required by Kirkpatrick’s training evaluation categories. We note that surveys lack the conceptual richness of qualitative data. An upscaled version of Ask the Specialist Plus is currently being delivered to three NT hospitals: this ongoing research program will be evaluated qualitatively with patient and healthcare provider interviews and observations and also quantitatively by monitoring self-discharge rates as a measure of cultural safety [25, 99]. 

## Conclusion

The Ask the Specialist Plus training program has shown promising results in supporting culturally safe communication in healthcare by fostering critical consciousness among healthcare providers. While there is still much work to be done to dismantle oppressive systems and improve racial literacy among healthcare providers, the Ask the Specialist Plus program has the potential to serve as a model for cultural safety and communication training for other jurisdictions. The program highlights the value of investing in novel pedagogical practices, the importance of privileging First Nations knowledges, cultures and voices and supports the premise that combating racism is a shared responsibility among racial groups. Results also highlighted that healthcare providers want to understand racism in healthcare so they can take action to dismantle it. By continuing to engage with and document experiences of racism, we can work towards reducing bias in healthcare and ultimately eliminating health inequities. The program’s focus on personal development and critical reflection has the potential to promote cultural inclusivity within healthcare settings, fostering a culturally safe hospital service, which is fundamental as a human right.

## Data Availability

Data from the study may be available from the corresponding author on reasonable request.

## Abbreviations

NT: Northern Territory
O and G: Obstetrics and Gynaecology department
RDH: Royal Darwin Hospital
WK: Week
